# Oral Tissue Interactions and Cellular Response to Zirconia Implant-Prosthetic Components: A Critical Review

**DOI:** 10.3390/ma14112825

**Published:** 2021-05-25

**Authors:** Marcel F. Kunrath, Saurabh Gupta, Felice Lorusso, Antonio Scarano, Sammy Noumbissi

**Affiliations:** 1 Dentistry Department, School of Health and Life Sciences, Pontifical Catholic University of Rio Grande do Sul (PUCRS), P.O. Box 6681, Porto Alegre 90619-900, RS, Brazil; marcelfkunrath@gmail.com; 2 Materials and Nanoscience Laboratory, Pontifical Catholic University of Rio Grande do Sul (PUCRS), P.O. Box 6681, Porto Alegre 90619-900, RS, Brazil; 3 Zirconia Implant Research Group (Z.I.R.G), International Academy of Ceramic Implantology, Silver Spring, MD 20901, USA; saurabh@iaoci.com (S.G.); sammy@iaoci.com (S.N.); 4 Master Dental Science, Universitat Jaume I, 12071 Castellón de la Plana, Spain; 5 Department of Innovative Technologies in Medicine & Dentistry, University of Chieti-Pescara, Via dei Vestini, 31-66100 Chieti, CH, Italy; drlorussofelice@gmail.com

**Keywords:** mucointegration, osseointegration, zirconia, biocompatibility, cell response

## Abstract

Background: Dental components manufactured with zirconia (ZrO_2_) represent a significant percentage of the implant prosthetic market in dentistry. However, during the last few years, we have observed robust clinical and pre-clinical scientific investigations on zirconia both as a prosthetic and an implantable material. At the same time, we have witnessed consistent technical and manufacturing updates with regards to the applications of zirconia which appear to gradually clarify points which until recently were not well understood. Methods: This critical review evaluated the “state of the art” in relation to applications of this biomaterial in dental components and its interactions with oral tissues. Results: The physico-chemical and structural properties as well as the current surface treatment methodologies for ZrO_2_ were explored. A critical investigation of the cellular response to this biomaterial was completed and the clinical implications discussed. Finally, surface treatments of ZrO_2_ demonstrate that excellent osseointegration is possible and provide encouraging prospects for rapid bone adhesion. Furthermore, sophisticated surface treatment techniques and technologies are providing impressive oral soft tissue cell responses thus leading to superior biological seal. Conclusions: Dental devices manufactured from ZrO_2_ are structurally and chemically stable with biocompatibility levels allowing for safe and long-term function in the oral environment.

## 1. Introduction

The requirements for biomaterials are for them to be biocompatible coupled with high durability while exposed to the harshness of the oral environment. Additionally, they should not affect or interfere with the recipient’s physiology and general health. Prosthetic components and implants made from zirconia (ZrO_2_) reveal excellent biological and mechanical properties and superior aesthetic advantages when compared to other biomaterials available on the market [1,2,3]. With the ever increasing body of research conducted around zirconia, clinical use of zirconia implants is on the rise due to their biological, aesthetic and physical properties. [4]. Moreover, it presents itself as an excellent material in the manufacture of customized implants, prosthetic components and various other dental prostheses by means of 3D printing technology [5,6,7].

The challenge with products manufactured with ZrO_2_ is their hardness and the complexity in the treatment of their surfaces [1,8]. However, current advanced manufacturing protocols have been able to develop nanoscale textures on this material by applying techniques such as anodizing, high-intensity lasers, acid etching and surface coatings [8,9,10,11,12]. Gnilitskyi and collaborators reported the use of high-speed femtosecond laser on ZrO_2_ surfaces for surface nanotexturization, which has been proven to be of significant importance in terms of cell adhesion and osseointegration in an animal model [9]. Thus, the nano-interaction between ZrO_2_-based surfaces and cells reveals a new and promising path in research which needs more scientific investigation.

Studies on the biological interaction of ZrO_2_ have become increasingly relevant and are following a path similar to other well proven materials such as titanium and its alloys [13,14]. Rottmar et al. demonstrated that zirconia surfaces had the best performance with regards to fibrinogen adsorption and thrombogenicity [15]. Furthermore, reports prove zirconia to have an advantage in terms of biological properties with soft peri-implant tissues thereby modulating fibers and cell attachment and behavior with greater effectiveness and biocompatibility [16,17]. Along with the properties mentioned above, zirconia has a low surface energy [18,19], therefore it retains very low amounts of plaque and consequently has less bacterial colonization on its surface. In a study, Kunrath et al. showed by comparing surfaces with different morphologies which were exposed to the bacterium *Staphylococcus epidermidis* that there was less bacterial adhesion on ZrO_2_ surfaces [18]. Moreover, Roehling et al., revealed a significant reduction in the formation of oral biofilm on zirconia surfaces after 72 hours [19].

With regards to clinical factors, implants made from ZrO_2_ demonstrate comparable osseointegration capabilities and clinical success to their metal alloy counterparts [4,20,21]. Zirconia ceramic implants inserted in New Zealand white mature male rabbits showed a bone-implant contact of 68.4% ± 2.4% with few narrow spaces and without presence of inflamed or multinucleated cells. This study concluded that these implants are highly biocompatible and osteoconductive [22]. Prosthetic components made of zirconia show resistance and durability to continuous cyclic loads as a result of chewing [20], in addition to revealing a better interaction with gingival tissues than metal alloys [14]. After 5 years of follow-up, no evidence of chipping or delamination of the ceramic veneering on the zirconia frameworks were observed when used for Toronto bridges [5]. Long-term randomized clinical controls have shown excellent soft tissue texture, quality and health when zirconia is used subgingivally and supragingivally [23]. Thereunto, this critical review shows a qualitatively improved level of interaction between oral tissues and ceramic dental components made from ZrO_2_. First, we will describe the treatment methodologies and the resulting surface characteristics and topographies; second, the influence of these surface variations on the immediate and late biological responses at the cellular level; and third, the relevant clinical implications of the use of these biomaterials.

## 2. Zirconia Applications and Variations

With the aim of offering an alternative to metal-based dental prostheses, structural ceramics have been improved and are now widely used in dentistry. Among all dental ceramics, zirconia has emerged as a versatile and promising material due to its biological, mechanical and optical properties which have contributed to its rapid and widespread adoption in dentistry. Zirconia has been a material of choice which, when used with CAD/CAM technology, has allowed the fabrication of various prosthetic components and customized implants for a broad range of treatment options. Zirconia-based ceramics are routinely used for structural applications in engineering such as in the manufacturing of cutting tools, gas sensors, refractories and structural opacifiers [24]. The ceramic composites that are currently in use in medical and dental devices originated from structural materials used in the aerospace and military industry. In order to meet structural demands, zirconia is doped with stabilizers to achieve high strength and fracture toughness [25]. These materials have been modified to suit the additional requirements of biocompatibility [26].

### 2.1. Structural Properties (Crystalline Phases and Stabilization)

Regarding the material properties, zircon is a grey-white metal mined from the earth, it occurs in nature and is a precursor of zirconium; whereas zirconia is an oxide of zirconium which is a ceramic. Zirconia does not occur naturally as a pure oxide, therefore it needs to be extracted from minerals such as zirconate (ZrO_2_-SiO_2_, ZrSiO_4_) and baddeleyite (ZrO_2_) minerals which are the major sources of zirconium. Zirconate is comparatively more abundant, but less pure, and hence requires significant processing in order to obtain zirconia [27]. Baddeleyite already contains high levels of zirconia ranging from 96.5 to 98.5% and therefore is known as a high purity source of zirconium metal and its compounds. Zirconium dioxide (ZrO_2_) resulting from baddeleyite, which is also known as zirconia, is a coarse oxide and presents as a monoclinic crystal structure at room temperature. However, it can be purified and processed at high temperatures and under high pressure to obtain a tetragonal or a cubic structure. The cubic structure is hard, optically flawless and translucent in appearance and is mostly used for making precious stones or gas sensors [26].

**Figure 1:**
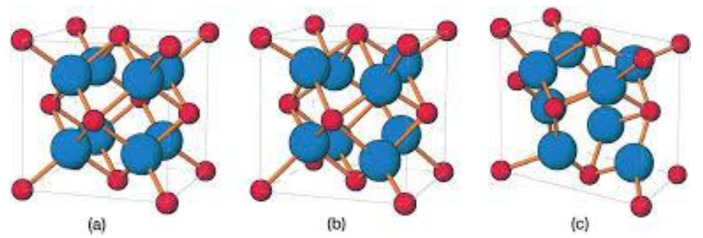
Scheme showing the 3 different atomic structures of ZrO_2_. Cubic (**a**); tetragonal (**b**) and monoclinic (**c**). Reprinted with permission from Wiley, reference [29].

Discrete crystallographic structures are seen in the spatial arrangement of atoms in zirconia, thereby representing a property called polymorphism. Zirconia has been classified based on specific geometry and dimensional parameters into three phases or crystal structures, namely, the monoclinic, tetragonal and cubic phases (Figure 1). Pure zirconia has a monoclinic structure at room temperature which is stable up to a temperature of 1170 °C. Between 1170 and 2370 °C, tetragonal zirconia formation takes place, whereas the formation of cubic zirconia is seen at much higher temperatures. After processing, depending on the cooling procedure used, the tetragonal phase reverts to the monoclinic phase at 970 °C. Since zirconia has polymorphic properties, pure zirconia cannot be used at elevated temperatures as large volume changes (3–5%) occur during the cooling process while transitioning to the monoclinic phase. This change is sufficient to go beyond the elastic and fracture limits, thereby resulting in the formation of cracks and flaws in the ceramic [28]. The transformation of the tetragonal phase to the monoclinic phase can be used to improve the tenacity like mechanical properties of zirconia. The mechanism involved in this process is known as a booster form transformation. This transformation is martensitic, and therefore, it is a process which occurs by shearing without diffusion, i.e., the change in atomic position occurs abruptly at a speed very close to the speed of sound propagation seen in solids. The reverse transition, i.e., the monoclinic to tetragonal transformation occurs at approximately 1170 °C, while the tetragonal to the monoclinic transformation that occurs during cooling, is observed between temperatures ranging from 850 to 1000 °C, depending on the strain energy. These structural changes make the manufacturing of components from pure zirconia impossible, due to the likelihood of spontaneous failure. The addition of stabilizing oxides is extremely important as it confers structural stability and maintains zirconia in its tetragonal form at room temperature [29]. To allow the tetragonal form to exist at room temperature after sintering, different oxides, such as yttrium oxide (Y_2_O_3_), calcium oxide (CaO) or magnesium oxide (MgO) are added to zirconia to stabilize it. The addition of different amounts of stabilizers also allows the formation of partially or fully stabilized zirconia which, when further combined with the changes in processes, may result in ceramics with high flexural strength/fracture toughness and excellent chemical resistance. A fully stabilized zirconia can be obtained by adding a sufficient number of stabilizing oxides, such as 16 mol% magnesia (MgO), 16 mol% limestone (CaO) or 8 mol% yttria (Y_2_O_3_). Since the partial stabilization of zirconia is also obtained with the same oxides, but in smaller proportions (like 2–3 mol% yttria), a multiphase structure is created, which usually consists of tetragonal and cubic zirconia in majority along with the monoclinic form precipitated in small amounts [27]. The transformation of tetragonal zirconia into its monoclinic form, which is also known as ageing, is a phenomenon that is influenced by temperature, vapor, particle size, micro- and macrostructure of the material and by the concentration of added stabilizing oxides. The particle size for the partially stabilized zirconia which is to be maintained in the tetragonal form at room temperature ranges from 0.2 to 1 μm (for compositions ranging from 2 to 3 mol% yttria), because, the transformation to the monoclinic phase is not possible when the particle size is less than 0.2 µm [25]. Thanks to material stabilization, zirconia has numerous medical and industrial applications. Zirconia is mainly used in bulk form or as a coating for implants/prosthetic devices, with the goal of greater surface resistance for the device, and biocompatibility.

#### 2.1.1. Monoclinic Zirconia

The natural form of zirconia, i.e., baddeleyite, contains approximately 2% HfO_2_ (hafnium oxide) which has very similar structural and chemical properties to zirconia. Zr^4+^ ions have a coordination number of seven for the O^2−^ ions that occupy the tetrahedral interstices, thus making the average distance between the Zr^4+^ ion and three of the seven O^2–^ ions, 2.07 Å. As the average distance between the Zr^4+^ ion and four O^2–^ ions in the structure is 2.21 Å, one of the angles (134.3°) differs considerably from that of the tetrahedral value (109.5°). Therefore, the structure of the oxygen ion is not planar but a curve is seen in the plane of the four oxygen ions, whereas a completely erratic plane is seen in case of the other three oxygen ions [29].

#### 2.1.2. Tetragonal Zirconia

In the tetragonal phase, zirconia appears as a straight prism with rectangular sides. Zr^4+^ ions show a coordination number of eight, but the shape once again gets distorted because of the fact that four oxygen ions are present at a distance of 2.065 Å in a tetrahedron plane, whereas the other four are at a distance of 2.455 Å in another tetrahedron plane which is elongated and rotated by 90° [30].

#### 2.1.3. Cubic Zirconia

The structure of cubic zirconia is represented as a simple cubic lattice with eight oxygen ions, surrounded by a cubic arrangement of cations, which is known as fluorite, i.e., the oxygen ions are present at the tetrahedral interstices of a cubic lattice structure (CFC) of cations [30].

## 3. Surface Modifications Aiming at Improved Biological Responses

### 3.1. Sand Blasting

Sandblasting, which is also known as airborne particle abrasion, produces a surface topography that has micro-roughness. Various parameters affect the roughness that is created on the implant surface, this includes the size, shape and kinetic energy of the particles used in the sandblasting process [31]. During the process of sandblasting, compressed air pressure creates an impulse which ejects the particles toward the surface of the implant. Thus, the kinetic energy which is obtained by the particles depends on their density, volume and velocity. The main advantage of the process of sandblasting is that a homogenous and gentle anisotropic abrasion can be obtained on hard materials like ceramic, glass and silicon. Alumina particles are the generally preferred sandblasting materials because of their low cost, hardness and needle-like shape. The major disadvantage of using the sandblasting technique is that it may slightly change the surface chemistry because of inevitable alumina contamination and in the case of ceramics induce micro-cracks within the implant or the prosthetic part prior to any functional stresses [32]. Many studies have proven that although the sandblasted zirconia surfaces show slight enhancement in cell attachment, their metabolic activity is still inferior to that of etched zirconia surfaces [31,33].

### 3.2. Acid Etching

The process of acid etching is performed with either hydrofluoric acid, nitric acid or sulfuric acid. Acid etching treatment can also be used to overcome alumina contamination as it has been proven to remove the alumina residues (Table 1). Heat treatment follows thereafter, which helps smoothen the sharp edges made as a result of the etching process [34]. Advantages of acid etching include the homogenous roughening of the material, regardless of its size and shape [35]. This method presents no risk of delamination and does not exert stress on the material [36]. However, it might cause undesirable chemical changes which can be a disadvantage of the process [37]. The topography formed after acid etching depends on prior treatment, composition of acid mixture, temperature and the length of exposure to the etchant. Acid etching is generally used to generate a micro scale surface texture which has the ability to achieve interlocking between the implant and the bone [35]. Recent studies show that combining the sandblasting and acid etching techniques enhances the degree of micro-roughness of zirconia as well. Such a combination has been proposed and is currently used in some commercially available zirconia implants; the purpose is to optimize micro-roughness, which would also provide a more receptive surface for osteoblast cell attachment and proliferation [34,38].

### 3.3. Selective Infiltration Technique

This technique involves coating the surface of the material with a specific infiltration glass and then heating it at a temperature higher than its glass transition temperature. This is followed by the infiltration of molten glass that occurs between the material grains (Table 1). This technique can be used for selective roughening because only the surface grains joined with the infiltration glass are involved in the process, thereby allowing control over the area requiring treatment. Traces of infiltration agent left behind, can further be removed by immersion in a solution of 5% hydrofluoric acid and rinsing with water [39]. This selective infiltration etching technique is often used to create a nano-porous surface on zirconia implants [40]. The major advantage of this technique is that the actual surface chemistry of material remains unchanged, and the nanoscale roughness of the surface can be enhanced without losing any material or changing the microscopic surface roughness.

### 3.4. Polishing

Polishing gives a comparatively smoother surface than acid etching and sandblasting (Table 1). It is known that the epithelial cells are more likely to adhere to rough surfaces (acid etching and sandblasting) as compared to smoother polished surfaces, whereas fibroblasts adhere well onto both roughened and smooth surfaces [41]. Polishing of a zirconia surface is performed by using a silicon carbide polishing paper and a diamond or silica suspension using a polishing machine [42]. Mechanical surface treatment that includes polishing, allows a change in the surface topography without modifying the actual surface chemistry or structural integrity. The average surface roughness of a polished zirconia biomaterial is between 8 and 200 nm [40,42]. Polishing also serves to clean the implant surface to a certain extent, along with giving it a smooth texture.

### 3.5. Laser Treatment

In contrast with sandblasting and acid etching techniques, laser treatment exerts zero risk of surface contamination as there is no direct contact between the laser and the biomaterial [43]. The laser surface treatments also tend to improve the material wettability by altering the surface properties, which further plays a major role in cell adhesion. The test for wettability is conducted by putting one drop of the liquid on a flat solid surface of the material and the contact angle is further used to represent the final shape of that drop (Table 1). The higher the contact angle, the lower the wettability of the material. The wettability of material also affects the protein adsorption and cellular adhesion [44]. Protein adsorption depends completely on the nature of the protein-bearing aqueous solution, and the cells are believed to behave differently in response to different organization of the adsorbed protein layers. It is therefore important that the implant surface wettability be elevated, thereby allowing optimal protein adsorption. However, the use of lasers on zirconia ceramic has been reported to disrupt its chemical structure with the potential of inducing pre-function cracks and partial transformation to the monoclinic phase as a result [44,45].

### 3.6. Ultraviolet Light Treatment

Various studies have shown that bone implant contact of the implants treated with ultraviolet (UV) light was deeply enhanced because of the effect of superhydrophilicity (Table 1). A material is described to be superhydrophilic when the contact angle of the water droplet is less than 5° [46,47]. Hydrophilicity is one of the key factors involved in the initial interaction with proteins and cells that is beneficial for the early phases of wound healing and osseointegration [48]. When the hydrophilic oxide surface binds to water, hydroxyl (OH^−^) and oxygen (O^2−^) groups are formed on the outermost layer. The formation of hydroxylated oxide surface improves the surface reactivity with the surrounding ions, amino acids and proteins in the tissue fluid. When compared to hydrophobic surfaces, osteoblasts cultured on hydrophilic surfaces have shown to exhibit higher levels of differentiation markers, including alkaline phosphatase and osteocalcin [49,50]. The effect of hydrophilic surfaces on osseointegration can be observed with improvements in the bone implant contact and bone anchorage that occurs in the early stages of bone healing. Other studies have shown that the atomic percentage of hydrocarbon will change after UV treatment [51,52].

### 3.7. Coating

Coating of yttrium stabilized zirconia (YSZ) with reinforced hydroxyapatite (HA) has shown positive results in the enhancement of adhesive strength and coating stability [50]. Because of their versatility, calcium phosphate (Ca(PO)_4_)(CP)-based coatings are generally fabricated using plasma-spraying techniques. Despite its numerous drawbacks, this technique is said to provide low cost and a high deposition rate [53,54] (Table 1). For depositing CP-based coatings, new techniques are constantly being developed to address the issues associated with plasma spraying. In an in vitro study, Pardun et al. synthesized a YSZ/HA coating by wet powder spraying (WPS) using a double action airbrush spray. Although WPS shows versatility in coating curved surfaces with different thickness, the long-term stability of these coatings has not been confirmed [55,56].

Because of its simplicity and highly versatile nature, electrophoretic deposition (EPD) has been proposed as an alternative to traditional techniques. An approach that combines both plasma electrolytic oxidation and electrophoretic deposition has been made to fabricate and coat zirconia or hydroxyapatite film on zirconium [57,58,59]. Good biocompatibility, corrosion resistance and bioactivity obtained through this process broadens the potential applications of EPD. Apart from HA, silica is also commonly used as a coating material for zirconia. The use of RKKP has also shown a positive cell response and osseointegration. Coating with RKKP was fabricated using either an enameling and firing technique or thermal treatment. AP40, another silica bioactive glass, was also determined to be comparable in terms of reactivity with RKKP [56]. Frandsen et al. sputter coated a commercial zirconia implant with a film of titanium and conducted anodization to produce a coating of TiO_2_ nanotubes on the surface. Along with the enhanced osteoblast behavior, superhydrophilicity was also observed on the treated implant [60].

### 3.8. Biofunctionalization

Biomimetic surface modification, also called biofunctionalization, involves immobilization of biomolecules on the surface to change their biochemical properties and biological responses [47]. Biofunctionalization also allows anchorage of organic components such as proteins, enzymes and peptides on the implant surface thereby determining the type of tissue that develops at the implant-bone interface (Table 1). Arginine-glycine-aspartate (RGD) is commonly used as an adhesive peptide [61]. Many other adhesive proteins are found to possess RGD as their cell recognition site, including fibronectin, fibrinogen and collagen. These RGD sequences are identified using at least one of the integrins [62]. Adhesion proteins and integrins form a pair to provide cell anchorage, differentiation and growth signal. The RGD peptide has shown to be successfully immobilized with physical-chemical modifications (surface polarization) or applying functionalized coatings on Y-TZP and thereby enhancing the biocompatibility of the material as well as cell attachment to its surface [61,63]. The existence of biomolecules at the surface of biomaterials simulates the native cellular micro-environment in control of cell behavior. For instance, Arg-Gly-Asp (RGD), a cell-binding order, which originated from the extracellular matrix (ECM) protein like the fibronectin, has been extensively used because it encourages cell adhesion by enabling integrin receptors. RGD-containing peptides improve the connection of numerous cell types on different types of biomaterials [61,63].

### 3.9. Self-Assembly

**Figure 2:**
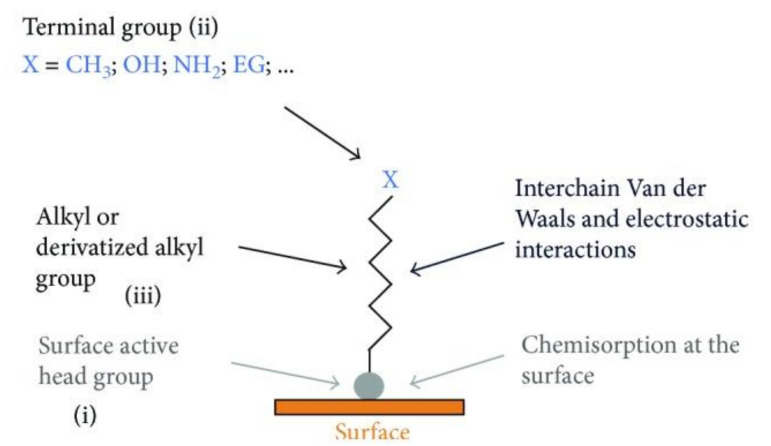
Scheme demonstrating the process of SAMs formation. Detailed chemical parts of the surface functionalization (i, ii and iii). Reprinted with permission under Creative Commons Attribution 4.0 International License, reference [65].

An autonomous process by which the components are organized into patterns or structures without any external intervention is called self-assembly [64] (Table 1). Self-assembled monolayers (SAMs) (Figure 2) are formed by the process of solution deposition, i.e., the immersion of a particular substrate into the solution of an active surfactant in a particular solvent which may either be organic or aqueous, or the solid surface vapor deposition of an active organic compound or by aerosol spraying. The driving force for self-assembly is generally the specific interaction between the head group of the surfactant and substrate surface. Most of the surfactants consist of three distinctive parts, namely, the surface active head group which binds strongly to the surface, the terminal group which is located at the monolayer surface shows the interfacial properties of the assembly and the alkane chain which serves as a linker between the head and the terminal groups and facilitates the packing of the molecules in the monolayer with the Van der Waals interactions between adjacent methylene groups that orient and stabilize the monolayer [65]. Therefore, by carefully composing the mixture of substrate, SAM solution, and subsequent terminal functionalization, a multitude of subsequent molecule adhesions is feasible [66,67,68,69]. In this typical procedure, clean or freshly prepared substrate is absorbed in a diluted 1/10 ml solution of surfactant compound(s) in highly pure solvent for 12–48 h at room temperature. After this, the slides are vacated, cleaned with solvent and dried under a flow of nitrogen [65,67]. Nanoscale modifications of dental implants have been an active scientific research area, where new techniques such as SAMs are offering approaches to standardize tissue response and microbiota micro environment in accordance with the conditions of bio-activity, anti-adhesion, anti-bacterial or combined effects (Table 1).

## 4. Biological Responses

**Figure 3:**
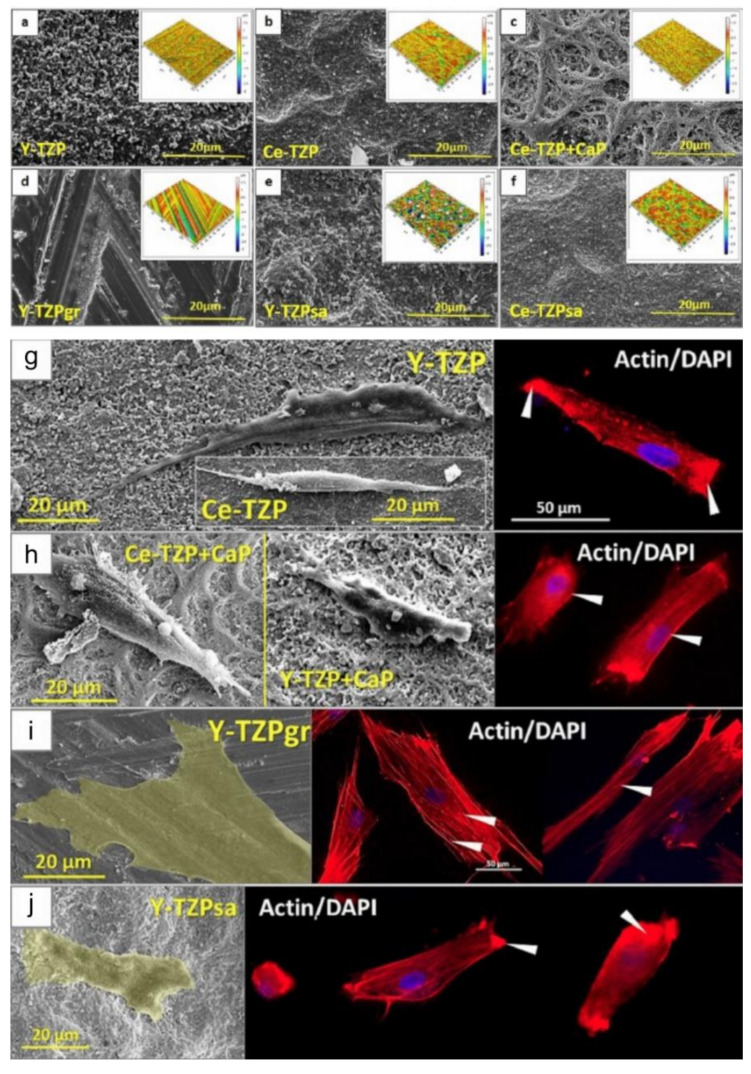
Different ZrO_2_-surfaces morphologies modified by varied surface treatments (**a**–**f**). Microscopies using electron microscopy (SEM) and confocal microscopy showing the different responses of osteoprogenitor cells regarding morphology and nuclei position on four different ZrO_2_-modified surfaces. The cells presented an elongated morphology in some surfaces (**g,h**) and a flattened morphology in other topographies (**i**,**j**). Showing, as reported in the study, that surface morphology characteristics in zirconia are more important for cell evolution and consequent proliferation than properties such as roughness and wettability. Reprinted and adapted with permission under Creative Commons Attribution 4.0 International License, reference [84].

**Figure 4:**
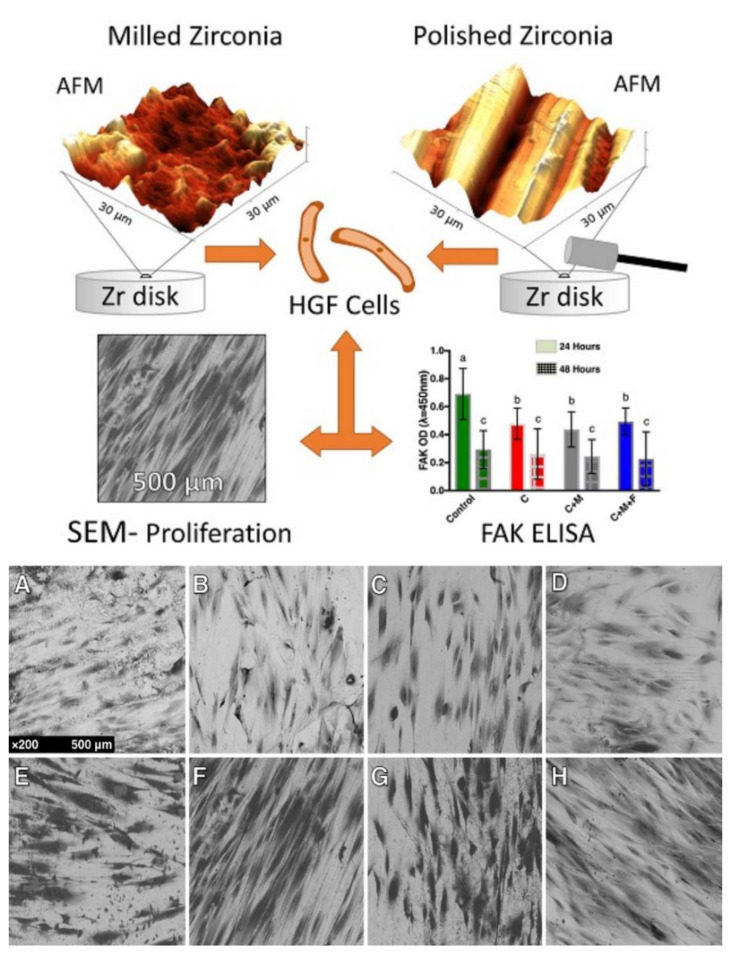
SEM micrographs demonstrating the different behavior and alignment of human gingival fibroblasts on different ZrO_2_-modified surfaces developed for implant abutments submitted to three different polishing protocols under a 24-h culture (**A**–**D**) and 48-h culture (**E**–**H**). The more polished surfaces showed greater cell counts (**B**–**D**, **F**–**H**) revealing an influence of polishing in the proliferation of this cell at 24 h and 48 h. Letters (a,b,c) represent statistical significance between the groups. Reprinted with permission under Creative Commons Attribution 4.0 International License, reference [88].

The biological responses performed after the placement of a biomaterial in the oral environment are very important for tissues where there is a need for healing [3,12,13]. Surface modifications, functionalization of ZrO_2_, as well as innovations in the base-material can induce individualized cellular responses promoting a better performance of the device once it is implanted [9,12,17,70]. As a result, intraosseous implants, components for implants and oral prostheses made from ZrO_2_ have attracted the attention of researchers and clinicians due to their biocompatibility properties (Table 2). Materials made from ZrO_2_ have consistently shown better biological reactions in periodontal and epithelial tissues where most of the cells are fibroblasts, red-blood cells, platelets, defense and epithelial cells [71,72,73,74,75,76,77,78,79,80,81,82,83,84,85,86,87,88,89,90,91,92,93,94,95,96,97,98,99,100]. In addition, these cells are crucial for the short and long-term success of biomaterials placed in the oral environment although their original task is to protect against contamination from the external environment. Active in vitro/in vivo research is being developed to discover the main ways to modify ZrO_2_ for better modulation of cells found in soft tissues (Figure 3 and Figure 4).

### 4.1. Blood-Surface Biological Interactions

During the process of installing a new intra or subgingival dental device, the first cell types to come into contact with the biomaterial are blood cells [71,72]. These cells have the ability to trigger a cascade of events that will stimulate the inflammatory and healing phases [72] (Table 2). Not only are red blood cells, white blood cells and platelets part of the process, but also several blood plasma proteins such as immunoglobulins, fibronectin, fibrinogen and vitronectin [71,72]. This set of cells and proteins is found in plasma and when interacting with the biomaterial, responds differently depending on the site of application and the different implant surface topographies [71,73,74]. Normally, proteins with high mobility are deposited first on the biomaterial surface and then replaced by proteins with less mobility, this process of sequential interaction of blood proteins is known as the “Vroman effect”. Under normal conditions, the deposition of blood proteins follows a well-established order (albumin, globulin, fibrinogen, fibronectin), however, changes in surface properties such as hydrophobia or hydrophilia, can change this order of adhesion and changes in surface energy can affect the speed at which the implant surface is exposed to blood proteins [71,72,73,74].

From this initial contact, the formation of a clot on the surface occurs as the first step in tissue healing [72]. The cluster of platelets and blood cells are induced from signals derived from the release of cytokines and other signaling molecules. This cascade of events promotes the development of a more intense vascularization at the target site, which induces the arrival of innumerable protective cells which also have a potential for differentiation. The migration and differentiation of new cells such as osteoblasts, mesenchymal cells and fibroblasts in the case of connective tissues take place at the interface of the biomaterial. Traini et al. compared three different surface morphologies, including machined Ti, microtextured Ti and microtextured Y-TZP samples. The study revealed a good response to fibrin adhesion and clot formation in zirconia samples, as well as better performance when the zirconia sample surface was compared with the machined Ti samples [71]. Comparing the blood-interaction between Ti and ZrO2 with similar roughness levels, the authors concluded that zirconia demonstrated less blood clot extension than microtextured Ti, and further studies should be conducted to further understanding of blood-interaction with different material surfaces [71].

Furthermore, the interaction with blood cells is not restricted only to bone tissues, some oral prosthetic components are inserted with their intra gingival structure where blood tissue can be found next to the epithelial tissue, inducing the same initial process of clot formation in the area [75]. On the other hand, before the formation process of new tissue, the cascade of events derived from scarring generates an inflammatory process when the new biomaterial is inserted. These steps are crucial to protect against infection and contribute to proper and efficient migration of tissue cells thereby inducing faster and protective scarring [72,74].

As a consequence, the acute process of inflammation develops, and cells such as neutrophils and leukocytes are present in significant quantities [76]. The release of histamine and mast cell degranulation are the first signaling events of this phase. As a result, phagocyte migration is induced by the presence of histamine in the region initiating the decontamination process, eliminating dead cells and inducing the proliferation of healing cells. The acute inflammatory process usually takes a maximum of one week, after which the migration of a new vascular network along with formation and maturation of collagen fibers. Moreover, the correct progress of the inflammatory process protects the biomaterial from being colonized by negative inflammatory cells such as giant cells or the dominance of cells that promote fibrous tissue growth. The interposition of this cell type in the superficial layer can interrupt the initial osseointegration process promoted by osteoprogenitor cells, generating a possible failure of bone adhesion in the subsequent healing phases. Thus, the correct combination of the presence of fibroblasts and differentiation of mesenchymal cells is of utmost importance for the growth of the new tissue on the surface of the biomaterial [74].

### 4.2. Osteoprogenitor Cells

The migration of osteoprogenitor cells to the sites where ZrO_2_ dental components are used is a determining factor in the success of these biomaterials in the oral environment. For example, the formation of a perfect bone crest is crucial for the success of both the implant prosthesis components as well as an intraosseous implant. Moreover, connective, periodontal and epithelial tissues depend on the underlying bone tissue for support and nutrition [77] (Table 2). Thus, undifferentiated cells, osteoblasts and osteoclasts must perform their functions when exposed to these new biomaterials.

Monru et al., through a comparison between ZrO_2_ treated with surface blasting and Ti treated with surface blasting, revealed a better performance in the spreading and migration of pre-osteoblasts on ZrO_2_ surfaces [78] (Table 2). In greater detail, Fernandes et al. evaluated the extracellular responses of pre-osteoblasts to ZrO_2_, their findings showed important changes in the extracellular arrangement because of contact with the zirconia surface. It was also determined that zirconia had a positive influence in the process of stimulating differentiation factors in cell phenotypes which play an important role in cell adherence and proliferation [79]. Similarly, authors [80] developed an anodizing methodology on ZrO_2_ surfaces and evaluated the cellular responses of MC3T3-E1 osteoprogenitor cells, as well as osteoclast changes. Their findings showed that the spread, proliferation and changes in cell morphologies are totally dependent on changes in the material’s surface. Furthermore, the same authors performed in vivo tests on rats, which demonstrated a better performance of the anodizing treatment in terms of osseointegration with high levels of bone apposition [80].

Animal studies demonstrate more conclusive results with regards to osseointegration. In rabbits, Mostafa and Aboushelib developed a bioactive surface (coated with nano-hydroxy apatite particle or platelet-rich plasma) on ZrO_2_ surfaces to evaluate the phenomenon in animal models. The developed surface showed excellent results in terms of the quality of osseointegration (histological bone apposition) when compared to the unmodified surfaces [81]. Likewise, Rezaei et al. [82] revealed, in rat femurs, that surfaces modified with innovative methodologies can increase osseointegration quality by up to two times when compared to untreated ZrO_2_. In addition, in vitro tests showed that the expression of genetic factors such as osteopontin, osteocalcin and BMP-2 was much greater for the treated surface [82]. Moreover, in a canine model, authors reported minimal peri-implant bone loss around zirconia implants after 4 weeks of functional loading [83] (Table 2).

The studies mentioned above reveal the significant role of the response of osteoprogenitor cells and mature bone cells when exposed to various zirconia surfaces. They also demonstrate that changes in micro- and nano-scale surface topographies can control cellular behavior, especially in the early stages of healing [84], stimulating the development of bone tissue and its adjacent soft tissues (Figure 3). Consequently, this combination of hard and soft tissue healing is essential for the short and long-term durability of zirconia ceramic dental components inserted in the oral environment.

### 4.3. Fibroblasts and Macrophages

For tissue growth and maturation adjacent to new components manufactured with ZrO_2_, proliferation of fibroblasts and collagen formation is necessary (Table 2). The differentiation and proliferation of these cells is decisive in the biological sealing process through the alignment and orientations of collagen fibers towards the new surface, developing and protecting the underlying tissue [83]. Therefore, components derived from ZrO_2_ must have an outer layer capable of inducing the differentiation of fibroblasts, as well as stimulating the formation of collagen which are essential in protecting against bacterial adhesion and infiltration. Authors suggested [16] that the superhydrophilic characteristic of zirconia is determinant for the adhesion and proliferation of gingival fibroblasts when compared to other materials. Research conducted by Tetè et al. showed that from a biological point of view, grinding and polishing zirconia surfaces seem to be advisable procedures because of the faster fibroblast response [101]. The fibroblasts’ interaction with a modified-ZrO_2_ structure can stimulate better cell behavior and performance resulting in stable and healthy soft tissue. Yang et al. [86], in a similar comparative study, revealed that the superficial functionalization of zirconia directly influenced the adhesion, proliferation and synthesis of collagen when using gingival fibroblasts thereby suggesting that superficial functionalization is significantly important as well as the chemical composition of the zirconia. Wang et al. showed that macrophages and fibroblasts have better cellular performance when in contact with hydrophilic surfaces, and that this property is more relevant than the chemical structure of the material [87]. In contrast, a study has shown that polishing zirconia surfaces manufactured by CAD/CAM stimulates greater proliferation and greater expression of gingival fibroblast adherence markers than rougher surfaces [88] (Table 2). Moreover, HGF-1 cells were found to not spread properly on micro-rugged ZrO_2_ surfaces, performing better on smooth surfaces [89]. Current systematic reviews have revealed the lack of in-depth information to compare the performance of dental components made of ZrO_2_ with regards to adhesion and soft tissue quality, suggesting the need for further preclinical studies in this area of research [90,91] This contrast of ideas and properties demonstrates that knowledge concerning the best surface treatment for zirconia to stimulate collagen cell/fiber adhesion and orientation is still not entirely clear, however, the results so far suggest promising clinical and physiological advantages of modified surface ZrO_2_ dental components (Figure 4) [92].

### 4.4. Epithelial Cells

Externally, the final layer of epithelial cells protects the underlying tissues and cells, as previously discussed, creating a thin and resistant layer that allows the formation of a totally new and complete tissue around the biomaterial inserted in the oral environment. The adhesion and proliferation of epithelial cells on the surface of dental components is of great importance for components inserted in areas of transition from the bone to the open oral environment. This is particularly important around healing and prosthetic abutments as well as dental crowns [91] (Table 2). Some formulations of zirconia samples showed greater cell viability and greater expression of intercellular contacts through hemidesmosomes when compared to smooth surfaces [94]. Nothdurft et al. revealed that the comparison between zirconia and titanium alloys results with regards to adherence of epithelial cells was without significant difference. However, when the surfaces are roughened there are significant declines in the adhesion of epithelial cells as rough surfaces are non-favorable to these types of cells [95]. Similarly, an in vivo study [96] in rats showed epithelial adherence similar for both Ti and ZrO_2_ implants inserted in the molar region. However, the in vitro analysis of the study demonstrated reduced levels of adhesion proteins on ZrO_2_ plates when compared to Ti, suggesting the need for further development of these surfaces for better epithelial healing [96].

Supporting this information, a coating composed of sol-gel-derived TiO_2_ was developed to improve the performance of human epithelial cells close to zirconia surfaces. The results revealed greater viability, proliferation and orientation of the cells when compared to surfaces without the coating [98], demonstrating the possibility of excellent results for transitional prosthetic components or dental zirconia implants. Moreover, current surfaces loaded with silver and gold nanoparticles are being tested to improve the entire healing process and stimulation of antibacterial responses [97].

The process of long-term durability of the implant and its prosthesis without the occurrence of pathologies such as peri-mucositis and peri-implantitis is mostly dependent on a mucosal seal provided by the gingival epithelial tissue and its adjacent tissues. A comparison in rats [99] revealed a stronger seal and a more adequate positioning of the formed tissue in order to prevent aggressors in ZrO_2_ implants. Furthermore, the expression of laminin-332 suggested regulating the elongation of the peri-implant mucosa, developing an improved biological area [99,100] (Table 2). This combination of results suggests a great potential for zirconia components in sites of epithelial transition, thereby providing more promising biocompatibility factors than the materials conventionally used.

### 4.5. Bacterial Cells

Many studies have shown both in experimental and clinical studies that plaque accumulation induces progressive bone loss around dental implants (Table 2). As a counterpoint, during the entire process of cell adhesion and healing, dental components inserted in the oral environment compete with bacteria both in supra-gingival and intra-osseous environments. This duel starts right in the first hours of contact of the surface of the new biomaterial with the environment in which they are placed and is also known as “the race for surface”.

However, a large portion of the intraosseous dental implants inserted in surgeries aiming at rehabilitation are aided by systemic antibiotics to fight infections [102]. On the other hand, transmucosal components, dental crowns, among other components, are usually not inserted along with this systematic protection, inducing the need for surface processes which stimulate the biological behavior of healing cells and inhibit the bacterial cells [102] (Table 2). Rough surfaces have historically shown greater potential for adherence of osteoprogenitor cells, inducing faster osseointegration in intraosseous devices, both in ZrO_2_ and in Ti [103]. However, the same characteristic is seen with regards to bacterial adhesion on surfaces inserted subgingivally or supragingivally [18,19]. Degidi et al. published an interesting study in humans where they found that the gingival inflammatory markers were high around titanium caps compared to the zirconium oxide ones [104]. In fact, the chemical composition and roughness parameters of healing caps have been shown to play an important role in the bacterial adhesion. Positively, some studies demonstrate advantages for surfaces derived from ZrO_2_ with regards to lower bacterial adhesion [105,106]. Roehling et al. demonstrated the lowest rate of bacterial adhesion after 72 h of anaerobic incubation on ZrO_2_ surfaces when compared to other surfaces with similar treatment [19]. Similarly, processes aimed at nanotexturing ZrO_2_ revealed a reduction in bacterial adhesion [107] (Table 2). In addition, authors reported [108] the development of a surface based on Ti-coated alloy with a combination of cubic stabilized zirconia and silver films, significantly reducing the adhesion of bacteria such as *Staphylococcus aureus* and *Staphylococcus epidermidis* which are responsible for peri-implant pathology.

Furthermore, relevant studies demonstrate that the main factors of bacterial adhesion to ZrO_2_ surfaces are its electrostatic state, as well as wetting properties [18,109] The interaction of chemical bonds between the extracellular membrane of bacteria and free radicals on the implant surface providess the necessary connection for the formation of a faster biofilm [18]. Thus, a great number of technologies have been researched and developed to combat the biofilm formed after installing the dental component in the oral environment as well as for cleaning and sterilization processes [18,110]. Methodologies such as laser therapy, photocatalysis, reactive plasmas and bio-electric effects are the most commonly tested today [110].

The processes that determine greater bacterial adhesion and proliferation are not yet fully understood. Much of the infection control is solved by the body’s defense cells against bacterial cells, thus the surface and chemical structure modification processes are additional protective mechanisms. There is a need for continuing development and research to improve dental component features in an environment where bacterial contamination is almost inherent.

## 5. Clinical Benefits

### 5.1. Osseointegration of Zirconia Implants

Osseointegration is one of the most important criteria for the success of an implant treatment. Bone apposition that takes place on different types of implant surfaces depends on the surface properties of the implant [111,112]. Authors suggested [113] that zirconia coating on the surface of titanium implants favors bone apposition more than titanium uncoated implants. The coating provides a favorable interaction between the bone-implant region promoting the development of more mature bone apposition [113].

Regarding post-loading osseointegration evaluation, Akagawa et al. [114], found that there is no significant difference in clinical features between the loaded and unloaded zirconia implants. However, the bone-implant contact for the unloaded group was 81.9% whereas it was 69.8% for the loaded group (Table 3). Another study that examined the role of osseointegration under various loading conditions around one-stage threaded zirconia implants, showed no difference in bone-implant contact ratio among the single freestanding, connected freestanding and mixed implant-tooth prosthetics using partially stabilized zirconia implants [115]. These findings were in agreement with a report that compared the bone-implant contact (BIC) (after 4 weeks of healing) of submerged zirconia implants, non-submerged zirconia implants and submerged titanium as the control [116] (Table 3). The results demonstrated the best performance with regards to bone-volume density in submerged zirconia implants (80%), followed by submerged titanium (74%) and non-submerged zirconia (63%) [116]. Moreover, no statistical difference was found between the BIC of all three types of implants when zirconia implants were compared to titanium and alumina [117]. Based on some studies, it was also suggested that the zirconia implants might withstand occlusal loads over a longer period of time [118].

During early healing, a similar rate of bone apposition on zirconia and surface-modified titanium implant surfaces was found during a histological examination of early bone apposition at 2 and 4 weeks after insertion [119,120]. No difference was seen in osseointegration between acid-etched zirconia implants and acid-etched titanium implants [119,120]. This was true even for the pharmacologically and chemically modified implant surfaces [38]. Another important property of zirconia dental material is their resistance to corrosion when exposed to the oral environment and body fluids. A recent review showed that zirconia oxide is highly resistant to corrosion, in fact titanium is known and proven to be vulnerable and susceptible to corrosion attack in the oral environment as opposed to ZrO_2_ [121].

### 5.2. Clinical Stability of Zirconia Implants

There are generally two types of modalities to assess osseointegration of dental implants. There are destructive methods such as the pull-push technique and reverse torque and on the other hand there are non-destructive modalities such as resonance frequency analysis (RFA) and the Periotest. It should be noted that none of those techniques and modalities measure osseointegration per se, they rather assess implant stability. The Periotest assesses stability by measuring the amount of micromovement of the implant and the RFA measures the frequency returning from an implant. In both cases, values are given to the measurements and correspond to a certain level of implant stability. Torque removal forces are used as a biomechanical measure for anchorage or osseointegration in which the greater forces required to remove implants may be portrayed as an increase in the strength of osseointegration [122] (Table 3).

In the study conducted by Sennerby et al. [123], it was found that coated zirconia and titanium implants showed higher removal torque values than the machined ones. The rabbit study conducted by Sollazzo et al. [113] showed that the titanium implants treated with zirconium oxide achieve a higher and statistically significant bone-implant contact percentage than in non-coated titanium implants. Bone was in direct contact with the surface of the zirconium dioxide implants, and the bone-implant interface was like that seen around Ti implants [124]. The findings suggested that the surface-modified zirconia implants can attain stability in bone. In another study where the removal torque values of various implants including the machined zirconia implants, sandblasted zirconia implants and acid-etched titanium implants were evaluated, machined zirconia had the lowest removal torque value whereas acid-etched titanium implants had the highest value, followed by sandblasted zirconia implants [123]. These findings suggest that the sandblasted zirconia implants can achieve a higher stability in bone than machined zirconia implants. Although it was seen that the coating of zirconia on titanium implants increased the removal torque value, in a study which compared the biomechanical properties of six types of implant surfaces, it was found in this study that removal torque value of zirconia implants was the lowest [125,126] (Table 3).

Hence, it can be concluded that the torque removal value for zirconia implants was surely increased after the process of surface modification but could not exceed that of titanium implants.

### 5.3. Clinical Cytotoxicity and Soft Tissue Response to Zirconia Implants

To test the biocompatibility of zirconia, various in vitro tests were conducted on osteoblasts, fibroblasts, lymphocytes, monocytes and macrophages where it was observed that zirconia had no cytotoxic effect on the bone forming cells and rather made them capable of elaborating the extracellular matrix by synthesizing various essential and structural proteins [74]. Zirconia is biocompatible as it does not induce any pseudo-teratogen effect [127] (Table 3). Laser-modified zirconia has shown a better adhesion to the bone forming cells due to their high wettability. Furthermore, it does not activate the pathologic inflammatory pathways as reported by Liagre et al. [127]. When tested with fibroblasts, wear products of zirconia friction showed cytotoxic only with a high percentage of particles release [128]. However, it has also been noted that further studies are required to provide any evidence. Both the powder and particle forms of zirconia when tested in vitro on different cell lines (human and murine) like lymphocytes, monocytes or macrophages, did not induce elevated cytotoxicity or inflammation compared to titanium [129] (Table 3).

During in vivo biocompatibility tests of zirconia, it was found that when it was implanted in the soft tissue, a thin layer of fibrous tissue encapsulated it, like what is seen with alumina. Furthermore, no cytotoxicity was observed in the soft tissue in relation to wear products of zirconia [130,131]. According to the findings of a study where pellets of stabilized zirconia with 6% Y_2_O_3_ were inserted into the femurs of monkeys, zirconia was found to be biocompatible to hard tissue when tested in vivo. As compared to alumina, no difference in bone reaction was seen in case of zirconia [132]. In a study by Kohal et al., it was found that cell proliferation around zirconia was comparable to that of titanium, but the surface modification of zirconia did not show any improvement in osseointegration [34].

Titanium implants have been recognized for many years as the gold standard in implantology. However, currently, a range of studies have been comparing the new possibilities of implants made from zirconia [133]. Tetè et al. found the collagen fiber orientation around zirconia implants to be similar to that of titanium, i.e., parallel to the implant surface [134]. According to van Brakel et al., zirconia had similar probing depth as titanium [135]. This finding was similar to the study finding of Kohal et al., wherein titanium and zirconia implants were put in the extraction sites of monkeys and both implants depicted the same peri-implant soft tissue dimensions [34]. Moreover, a study found that titanium had more mucosal color change as compared to zirconia [136], which was further contradicted by Zembic et al. [137] (Table 3). According to a study by Kajiwara et al., the blood flow in tissue surrounding zirconia abutments is similar to that in soft tissue around natural teeth [138] (Table 3) and better than around titanium abutments. Animal studies by Liñares et al. have demonstrated a higher degree of soft tissue integration around zirconia implants compared to that for titanium implants [139].

These comparisons between the different biomaterials for implant development show the intense evolution of materials derived from zirconia with promising results to achieve the results observed with the gold standard.

### 5.4. Limitations

In recent years, application of dental components derived from ZrO_2_ have seen increased application in dental clinics, largely due to technological developments in relation to treatments and changes in the properties of this material. One of the main restrictions on the use of zirconia was its high difficulty in being superficially treated in order to have a period of rapid osseointegration [8,14]. However, current studies show that this limitation is being overcome with extensive techniques of nanotexturization and functionalization of ZrO_2_-based surfaces [8,9,140]. Clinically, the use of implants with a single body requires extreme ability by the professional for a correct three-dimensional positioning without prejudice for subsequent prosthetic manufacture [141]; technological details that are also being perfected by companies with the manufacture of implants with separate parts [142]. Finally, corrections in components made with ZrO_2_ after the period of osseointegration or cementation are extremely difficult due to their hardness and the possibility of creating cracks with the use of specific burs [143]. Thus, the use of dental materials derived from zirconia must be carried out with a correct learning curve and planning by the clinical professional.

## 6. Conclusions and Future Challenges

This critical review explored the main physico-chemical and biological aspects that influence the healing process and adaptation of dental components manufactured with ZrO_2_. Many methodologies have evolved towards the design of micro- and nano-topographies in attempts to elicit optimal cellular response and behavior. In addition, many in vitro and in vivo studies reveal advantages for ZrO_2_ with regards to the interaction with soft tissue cells such as fibroblasts, blood cells and epithelial cells; the same applies to proteins adsorption, cells alignment and biocompatibility.

Clinically, ZrO_2_ surfaces showed excellent bone apposition and elicit better, or at least, similar, soft tissue responses comparing with other recognized dental materials for oral rehabilitation, demonstrated over time under appropriate conditions and when undergoing similar or identical surface treatment. Advantages in relation to periodontal seal are demonstrated by better attachment and alignment of collagen fibers with components developed in ZrO_2_. Less bacterial adhesion on smoother zirconia ceramic components, mainly prosthetic components, has been demonstrated and proven by a large range and number of reports. The prospects for a broader range of components made from ZrO_2_ have become increasingly promising for all areas of oral rehabilitation such as intra-bone, transmucosal and supra-gingival components.

Nevertheless, the constant technological evolution of the surface treatment of materials as well as in their macro, micro and nanoscopic design, reveal the need for intense research with clinical controls for a safe and predictable application of innovative materials in the market. At this stage it is not possible to clearly determine which combination of surface properties of zirconia performed the best at all cell levels, it is still unclear and remains a challenge for researchers and clinicians. To solve this challenge there is promise of multifunctional surfaces (with characteristics that stimulate positive cell proliferation and combat bacterial cells). Thus, more studies are needed to improve bioactive surfaces for supra and subgingival implantable ceramics.

## Figures and Tables

**Table 1:** Summary of the current chemical and physical treatments for zirconia implant surface.

Zirconia Implants Surface Treatments				
Treatment	Procedure	Disadvantages	Characteristics	References
Sandblasting	High pressure alumina (Al_2_O_3)_ release	Surface micro-cracks, Structural stress, contaminations	Low cost, hardness and needle-like shape	[31,33]
Acid etching	Combinations of: (1) ≃48% hydrofluoric acid (HF) (2) ≃70% nitric acid (HNO_3_) (3) ≃98% Sulfuric acid (H_2_SO_4_)	Undesired chemical changes	Remove the alumina contamination. Micro scale surface texture for bone to implant contact interface	[35,36,37]
Selective infiltration technique	Coating and glass heating procedure	Extended only to the surface grains	Nano-porous surface	[39,40]
Polishing	Silicon carbide polishing paper with diamond or silica suspension	Smoother surface compared to acid etching and sandblasting	Average surface roughness between 8 and 200 nm. No surface chemistry modifications.	[40,41,42]
Laser treatment	(1) CO_2_ laser (2) ER:YAG (3) Cr:YSGG	Disrupts chemical structure	No surface contamination. Improve material wettability	[43,44,45]
Ultraviolet light treatment (UVC)	UVC photons	No effects on surface roughness and surface chemistry	Effect of superhydrophilicity	[46,47,48,49,50,51,52]
Coating	Obtained by electrophoretic deposition (EPD) and plasma-spraying: (1) Reinforced hydroxyapatite (HA) (2) Calcium Phosphate (Ca(PO)_4_) (3) Bioglaze (RKKP)	Coating-implant bond strength and modification of chemical structure	Low cost and a high deposition rate. Good biocompatibility, corrosion resistance, and bioactivity	[53,54,55,56,57,58,59,60]
Biofunctionalization	(1) Immobilized arginine—glycine—aspartate (RGD)	Structural chemical changes	Improved biochemical properties and biological responses	[47,48,49,50,51,52,53,54,55,56,57,58,59,60,61,62,63]
Self-assembly	Self-assembled monolayers of active organic compound and terminal functionalization	Van der Waals layer interactions	Surface vapor deposition of active organic compound and molecule adhesion	[64,65,66,67,68]

**Table 2:** Summary of the current cellular and tissular interactions of the ZrO_2_ derivates.

ZrO_2_-Derivates Interactions				
Cellular and Tissular Response	Tissue	Cells	Effects	References
	Connective tissue cells	Fibroblasts Macrophages	-Increased cells migration and proliferation. -Fibronectin and vitronectin release. -Collagen and extracellular matrix proteins release. -Better cellular activity with hydrophilic surfaces.	[85,86,87,88,89,90,91,92]
	Blood cells	Erythrocytes Platelets	-Fibrinogen cascade activation. -Plasma proteins activation.	[71,72,73,74]
	Defense cells	Neutrophils, Leukocytes	-Histamine release. -Mast cell degranulation.	[71,72,73,74,75,76,77,78,79,80,81,82,83,84,85,86,87,88,89,90,91,92,93,94,95,96,97,98,99,100,101]
	Epithelium tissue	Epithelial cells	-Increased differentiation and proliferation. -Faster healing process and protective scarring.	[93,94,95,96,97]
	Osteoprogenitors	Osteoblasts	-Increased migration and proliferation. -Increased activity of osteopontin, osteocalcin, BMP-2 genes.-Osteoprogenitors sells adherence and proliferation.	[77,78,79,80,81,82,83,84]
	Oral biofilms cells	Bacteria cells	-Lower bacterial adhesion and proliferation. -Reduced bacteria activity.	[102,103,104,105,106,107,108,109,110]

**Table 3:** Summary of the hard tissues’ response of the ZrO_2_-based materials.

Bone Tissue Response to ZrO_2_			
Effect	Author	Effectiveness	Reference
Implant Loading	Akagawa et al. Stadlinger et al.	No bone-implant contact (BIC) with significant difference between the loaded and unloaded zirconia implants (BIC loaded: 81.9%; BIC unloaded: 69.8%). No BIC significant difference submerged zirconia and the non-submerged zirconia implants.	[114,116]
Chemical Property	Gahlert et al. Noumbissi et al. Sollazzo et al.	No difference of bone formation pattern in direct contact with zirconia and surface-modified titanium implant surfaces. Zirconia oxide high resistance to corrosion and ions release. Higher BIC percentage of zirconia implant compared to titanium implant.	[113,119,121]
Surface Treatments	Sennerby et al.	Sandblasted zirconia implants can achieve a higher stability in bone than machined zirconia implants.	[123]
Biocompatibility	Liagre et al. Hisbergues et al. Helmer et al.	No pseudo-teratogen effect. No evidence of high cytotoxicity or inflammation. No evidence of local bone reaction associated to the alumina treatment.	[127,129,132]

## Data Availability

Data sharing not applicable.
